# Editorial: Outcome Measures to Assess the Benefit of Interventions for Adults With Hearing Loss: From Research to Clinical Application

**DOI:** 10.3389/fnins.2022.955189

**Published:** 2022-08-18

**Authors:** Isabelle Boisvert, Melanie Ferguson, Astrid van Wieringen, Todd Andrew Ricketts

**Affiliations:** ^1^Sydney School of Health Sciences, Faculty of Medicine and Health, The University of Sydney, Camperdown, NSW, Australia; ^2^Curtin enAble Institute, Faculty of Health Sciences, Curtin University, Perth, WA, Australia; ^3^Brain and Hearing Research Group, Ear Science Institute Australia, Perth, WA, Australia; ^4^Research Group Experimental Oto-Rino-Laryngologie, Department of Neurosciences, University of Leuven, Leuven, Belgium; ^5^Maddox Memorial Hearing Aid Research Laboratory, Department of Hearing and Speech Sciences, Vanderbilt University Medical Center, Vanderbilt University, Nashville, TN, United States

**Keywords:** outcome measures, hearing loss, adult, clinical application, intervention

Hearing, listening, communication and participation in the context of hearing loss are complex constructs to measure. This is because those constructs are intertwined with other complex constructs including language, cognition, social engagement, and fatigue. Hearing loss (passive) impacts listening (active) which, for many adults who live with hearing loss, impacts communication (bi- or multi-directional exchange) and participation (everyday life). A plethora of hearing-based interventions are available to support the needs of adults with hearing loss. This includes a range of hearing aid and hearing implant technologies, personal sound amplification products, assistive-listening devices, communication strategies, and auditory/cognitive training. To evaluate the benefits of these interventions, we require valid, relevant and reliable outcome measures before and after the interventions.

A valid outcome measure means that it measures what it intends to measure, a relevant measure is that which taps into the intended mechanism of benefit or outcome domain, while a reliable measure means that the same result would be found if that measure was repeated in the same circumstances (high test-retest reliability). Because of their simplicity and reliability, hearing thresholds and speech recognition tests, typically conducted at a fixed volume in a quiet environment, have dominated clinical practice and research in audiology (Granberg et al., 2014). Although replicable, simple to conduct, and useful in certain contexts, these measures have limited relationship with everyday abilities and needs (Ferguson et al., 2016; Keidser et al., 2020). Developing and selecting valid, relevant and reliable outcome measures remains a challenge in the field of audiology.

We may never succeed in developing a single measure that captures the full relationship between hearing, listening, language, cognition and participation in the context of interactive and sustained communication within the real-world: a dynamic 3D acoustic and visual environment. However, it is important that we explore, and push, the boundaries of this problem in order to benefit the field, and importantly, better support those who live with hearing loss. The collection of articles in this Research Topic highlights current discussions and directions that the field has, and is, taking. This Research Topic begins with a scoping review by Neal et al. that maps the abundance of measures that are used in recently published studies to assess the listening and communication skills of adults with hearing loss. The authors note that these measures mainly target a narrow set of relevant domains. Following from this, Munro et al. discuss how the selection of hearing-related measures that are used in clinical trials have consequences on the outcomes of those trials—and therefore the knowledge that we can derive from these studies. Their article provides guidance about the factors that need to be considered in the development and selection of outcome measures, to increase the value and impact of clinical trials. The article by Allen et al. provides further reflection about the need to carefully consider the choice of self-report outcome measures used, not just for specific clinical trials, but in the development of national databases. For this to be possible, mechanisms are required to standardize the selection, collection and reporting of clinical data. These authors report on a consensus-based approach used to identify a core outcome domain set that is relevant to measure from the perspective of hearing services consumers and clinicians. Aligned with some of the core messages of Munro et al. and Allen et al., Dietz et al. illustrate how selecting the type and the timing of outcome measures impacts a study's outcomes, in particular the additional value that self-report measures provide over and above the conventional speech testing for cochlear implant users. Similarly, Abdel-Latif and Meister highlight how the addition of measures of listening effort can complement routine clinical testing. Hoppe et al. further demonstrate how considering outcome measures for each individual ear, as well as binaurally, impacts the interpretation of study results, in particular for individuals who have asymmetric hearing. In contrast with the numerous studies that use pure-tones and speech-based stimuli, Shafiro et al. conducted a systematic review to showcase the limited evidence base that exists in relation to the perception of environmental sounds with hearing devices. As for the abovementioned studies, inconsistent methodologies limit the potential to compare between studies and to aggregate data from larger datasets, for example for meta-analyses.

To assess outcomes that better reflect real-life situations, the complexity and realism of the stimuli and tasks can also be varied (e.g., using overlapping stimuli types, multiple stimuli locations, or dual-tasks). Historically, the main problem with these types of measures is that they have required larger spaces and more complex and expensive equipment. These measures also need to be designed and evaluated carefully to ensure adequate reliability (Ferguson and Henshaw, 2015). In this Research Topic, Miles et al. assessed new speech intelligibility tasks that are more representative of everyday speech communication outside the laboratory. They show that the more realistic speech task offered a better dynamic range for capturing individual performance and hearing-aid benefit across a range of real-world environments. The article by Salorio-Corbetto et al. describes the assessment of a Virtual Acoustics (VA) version of the Spatial Speech-in-Noise (SSiN) test, the SSiN-VA, for the purpose of evaluating hearing abilities with bilateral hearing aids. This approach can enhance clinical efficiency because testing can be conducted at home. In a similar vein, van Wieringen et al. investigated three different speech perception assessments in the same 40 cochlear implant users in their home environment. Their study showed that home-based speech perception testing is reliable and can be used to complement care in the clinic.

Outcome measures relevant for adults with hearing loss can be categorized in terms of the type of responses collected from participants (behavioral, physiological, or self-reported). In this Research Topic, however, no articles investigating physiological measures were submitted. In contrast, several submissions included self-reported measures, which are easy to conceptualize in terms of validity and relevance. While Neal et al. identified 139 different self-reported measures used with adults with hearing loss in recently published studies, self-reported measures continue being developed. Specific techniques for the development of high-quality Patient-Reported Outcome Measures (PROMs) have been developed, as described in the article by Laplante-Lévesque et al. Modern PROMs are therefore expected to include the rich perspective of people with the lived experience of the construct being measured, and follow good practice guidelines (e.g., COSMIN). Using the Cochlear Implant Quality of Life (CIQOL) instruments as an example, these authors provide useful context and guidance for research groups interested in using existing, or developing new self-reported outcome measures. In terms of new patient-reported measures, Humes' contribution describes the development of a new scale to measure the Subjective Wellbeing of older adults with hearing loss. Tapping into the domains of Life Satisfaction, Acceptance of Hearing Loss, and Social Support, the psychometric analysis of this new scale showed very good reliability and good criterion validity. Another new self-reported measure in this Research Topic and presented by Markodimitraki et al. is the COMPASS PROM that aims to quantify the consciousness of wearing a cochlear implant and how this impacts the daily life of cochlear implant users. This includes sleep disturbances due to the physical sensation of the implant on the head or problems with wearing headgear.

Acknowledging the progress made, as well as the need to select outcome measures that are aligned with specific research questions, more work is required before we can agree on an integrated set of outcome measures that are valid, relevant and reliable to support the everyday communication of adults with hearing loss. Study results based on such sets of outcome measures are critical when policy makers approve and fund new products and services. Therefore, with the inclusion of hearing benefit claims within the advertising of everyday technologies such as earphones, the development of alternative services delivery models (e.g., remote, automated, over-the counter, direct-to-consumer), the proliferation of hearing-related training programs, and the development of drugs that aim to improve hearing, the need for valid, relevant and reliable outcomes measured cannot be understated, and their selection cannot be overlooked.

## Author Contributions

IB wrote the initial draft of this article and all authors listed made a substantial, direct, and intellectual contribution to the work and approved it for publication.

## Funding

AW was supported by the KU Leuven grant no. C3/21/046.

## Conflict of Interest

The authors declare that the research was conducted in the absence of any commercial or financial relationships that could be construed as a potential conflict of interest.

## Publisher's Note

All claims expressed in this article are solely those of the authors and do not necessarily represent those of their affiliated organizations, or those of the publisher, the editors and the reviewers. Any product that may be evaluated in this article, or claim that may be made by its manufacturer, is not guaranteed or endorsed by the publisher.
